# Are parents' knowledge and practice regarding immunization related to pediatrics’ immunization compliance? a mixed method study

**DOI:** 10.1186/1471-2431-14-20

**Published:** 2014-01-25

**Authors:** Omer Qutaiba B Al-lela, Mohd Baidi Bahari, Harith Khalid Al-Qazaz, Muhannad RM Salih, Shazia Q Jamshed, Ramadan M Elkalmi

**Affiliations:** 1 School of Pharmacy, Faculty of Medical sciences, University of Duhok (UOD), Duhok, Iraq; 2 Faculty of Pharmacy, AIMST University, Kedah, Malaysia; 3 College of Pharmacy, University of Mosul, Mosul, Iraq; 4 Pharmacy Department, Al-Rashed University College, Baghdad, Iraq; 5 International Islamic University Malaysia, Kulliyyah of Pharmacy, Pahang, Malaysia

**Keywords:** Immunization, Iraq, Children, Parents, Knowledge, Practice, Compliance

## Abstract

**Background:**

Immunization rate is one of the best public health outcome and service indicators of the last 100 years. Parental decisions regarding immunization are very important to improve immunization rate. The aim of this study was to evaluate the correlation between parental knowledge-practices (KP) and children's immunization completeness.

**Methods:**

A mixed method has been utilized in this study: a retrospective cohort study was used to evaluate immunization completeness; a prospective cross-sectional study was used to evaluate immunization KP of parents. 528 children born between 1 January 2003 and 31 June 2008 were randomly selected from five public health clinics in Mosul, Iraq. Immunization history of each child was collected retrospectively from their immunization record/card.

**Results:**

About half of studied children (n = 286, 56.3%) were immunized with all vaccination doses; these children were considered as having had complete immunization. 66.1% of the parents was found to have adequate KP scores. A significant association of immunization completeness with total KP groups (p < 0.05) was found.

**Conclusions:**

Future efforts are required to improve immunization rate and parents' knowledge and practice. The study results reinforce recommendations for the periodic assessment of immunization rate and the use of educational programmes to improve the immunization rate, knowledge and practice.

## Background

Parental decisions regarding immunization are very important for increasing the immunization rate and compliance and for decreasing any possible immunization errors. Parents’ knowledge and practices regarding immunization are the major factors that contribute to their vaccination decisions [1].

There are many barriers against immunization, including misinformation about vaccines, adverse effects of vaccines, vaccine-preventable diseases, and disease development after the administration of vaccines [1-3]. Deficiencies in parents’ knowledge about the adverse effects and contraindications of vaccines often lead to many immunization errors. Many parents believe that mild illness is associated with vaccine contraindication, therefore mild illness is considered as a reason for not giving their children up-to-date vaccinations [4-6].

To improve parents’ awareness, good knowledge regarding vaccination is required. Therefore, physicians, pharmacists, nurses, and others health care providers should provide parents with correct information about the risks and benefits of vaccines [7]. Many studies showed that parents’ knowledge regarding child immunization varies according to the family physician and other medical staff [8-10]. Although parents would like to know about the adverse effects, the benefits and other information about vaccines, many physicians include vaccine risk in their discussions with parents without comparing it to the risks involved in infectious disease [11].

Good parental practice regarding immunization will be able to reduce the incidence of infectious diseases. Parental practice regarding vaccination is related to appropriate sources of information, the number of sources, and the way that vaccine information is received by parents. The sources of information provided by maternity clinics, the media, literature, and the internet cover vaccination benefits and the risks of vaccine-preventable diseases [12,13].

The most important factor affecting parental practice is communication between parents and the sources of information or immunization providers. Improving communication will improve parents’ perceptions of the benefits and risks of vaccines [14-18]. Parents will be more likely to continue with their child’s immunization, although at the same time they may still be doubtful about vaccination. In addition, parents may agree to proceed with their child’s vaccination, but they are also vulnerable to competing sources of information from anti-immunization proponents. This does not always mean that parents possess the knowledge and practice that constitute informed consent prior to assenting to immunization.

Most of the previous studies found a strong relationship between paediatric immunization coverage and parental knowledge and vaccination practices. This relationship showed a positive correlation between these factors. In other words, any increase in parental knowledge and practice will lead to increases in vaccination rates of children [19-24]. This study will provide a clear information regarding immunization in Iraq and parent knowledge- practice on paediatrics’ immunization.

## Methods

An observational non-experimental cohort study design was utilized to evaluate immunization rate among children younger than 2 years of age (born between 1 January 2003 and 31 June 2008). Each child had an immunization card for recording details of the immunizations received. A prospective cross-sectional study design was used to determine parental immunization knowledge and practices in Iraq, where the data were collected through a developed and validated questionnaire via interviews [18].

This study only covered the types of vaccines administered before 2 years of age: Bacille Calmette-Guérin (BCG) vaccine, oral polio vaccine (OPV), diphtheria-tetanus-pertussis (DTP) vaccine, hepatitis B virus (Hep B or HBV) vaccine, measles-mumps-rubella (MMR) vaccine, and the measles vaccine. A child was considered up to date if the following immunizations had been received by 2 years of age: one BCG dose, five polio vaccine doses (OPV), four DTP vaccine doses, three HBV vaccine doses, and one MMR vaccine dose.

The immunization status of the children was classified into two groups depending on immunization completeness: *complete immunization* and *partial immunization*. When a child received all immunization doses, this child was considered to have had *complete immunization.* If a child missed at least one immunization dose, then this child was considered to have had *partial immunization*.

The assessment of immunization knowledge and practices by developed and translated questionnaire was adapted from previous study [18]. The immunization knowledge and practices questionnaire consisted of 20 (10 questions on knowledge and 10 questions about practices) single-choice questions from a multiple answers provided in each equation, as shown in Table 1.

**Table 1:** Correct and incorrect answer

**Statements**	**Frequency (%)**	
	**Correct answer**	**Incorrect answer**
1 Vaccination prevents disease.	447 (84.7)	81 (15.3)
2 Vaccination is for all ages.	187 (35.4)	341 (64.4)
3 There are different types of vaccines	374 (70.8)	154 (29.2)
4 Active immunization is a killed or weakened form of a disease-causing agent.	341 (64.6)	187 (35.4)
5Passive immunization is an antibody from someone who was infected with the disease.	243 (46.0)	285 (54.0)
6 In some health situations, vaccines should not be given.	431 (81.6)	97 (18.4)
7 Vaccines need to be stored at more than 8 degrees Celsius and do not freeze.	206 (39.0)	322 (61.0)
8 The product should be used within 72 hours of the seal being broken.	56 (10.6)	472 (89.4)
9 There is a uniform immunization guideline for paediatric patients younger than two years.	343 (65.0)	185 (35.0)
10 Vaccination is harmful.	301 (57.0)	227 (43.0)
11 Are you in favour of vaccination?	496 (93.9)	32 (6.1)
12 Will recommend vaccination to others.	507 (96.0)	21 (4.0)
13 Vaccination should be initiated in the first week of age.	451 (85.4)	77 (14.6)
14 Were you informed about vaccination?	409 (77.5)	119 (22.5)
15 Did you read about vaccination in the media?	314 (59.5)	214 (40.5)
16 Did you see a television programme about vaccination?	367 (69.5)	161 (30.5)
17 Did you hear about vaccination on the radio?	214 (40.5)	314 (59.5)
18 Did you read about vaccination on the internet?	125 (23.7)	403 (76.3)
19 Did you obtain information about vaccination from an antenatal clinic?	348 (65.9)	180 (34.1)
20 Did you obtain information about vaccination from a maternity hospital or home?	212 (40.2)	316 (59.8)

Scoring of the questions was determined by giving one point (1) for each correct answer and zero (0) for wrong answers or no response (don’t know). The total knowledge scores and practice scores of the parents were calculated by adding up the scores for each question in the test. The total knowledge and practice scores ranged from 0 to 20, with higher scores indicating a higher level of immunization knowledge and practices. According to the median split method [25-27], parents with a total score of less than 12 (median) were considered as having inadequate knowledge and practices regarding child immunization and parents with scores from 12 to 20 were considered as having adequate knowledge and practices. This scoring method and categorization were used to identify the degree of parental immunization knowledge and practices in the current study.

According to the Iraqi Ministry of Health Survey in 2008, a total of 116,000 children were to be immunized in Mosul [28]; the present study used this number as the total population from which the sample size was drawn. An automated software program (Raosoft sample size calculator for study: http://www.raosoft.com/samplesize.html) was used to calculate the sample size required for this study. With an accepted margin of error of 5% and a 95% confidence interval, the sample size required was 383. With the addition of 30% (as expected drop outs) to the estimated sample size in order to overcome erroneous results and increase the reliability of the results and the conclusion, the target sample size was 500 children.

Most of the Iraqi children received their immunization doses from general and public health clinics. Cluster sampling method was used in this study. Mosul divided to five parts or clusters depending on the Mosul map. One health clinics selected from each cluster in Mosul (five health clinics from five different area or clusters). These public health clinics operate three immunization days per week (Sunday, Tuesday, and Thursday), from 9.00 am. to 2.00 pm. Approximately 25 children attend these health clinics per day. Private clinics and hospitals were not included in the setting for this study due to accessibility problems and differences in socioeconomic status.

The research proposal was submitted to the Iraqi Ministry of Health (MOH) in Baghdad, Iraq. Approval from the MOH (Reference no. 70667 in 15/12/2009) was obtained to facilitate the data collection by researchers from health clinics under Iraqi Ministry of Health before data collection was started. The parents and clinic staff were informed about the study aims and other details. The parents who were agreed to participate in the research had to sign the provided consent form before filling in the questionnaire. Most of the Iraqi children received their immunization doses from general and public health clinics. Five health clinics in different areas were selected in Mosul city, Iraq.

The data were analysed using SPSS for windows (Statistical Package for Social Science) version 20 and the level of significance was set at less than 0.05 for all analyses. Study samples were non-normal distributed, Mann–Whitney U test and Chi-square test were used in this study to evaluate the difference and association of KP among and with difference groups, respectively.

## Results

A total of 528 children and parents were recruited randomly in this study. Two hundred and eighty-sex children were immunized with all vaccination doses (56.3%); these children were considered as having had complete immunization, but less than half of the children had one or more than one missed doses and were considered as having had partial immunization.

The total knowledge-practice scores ranged from zero to 20 and the result showed an average of 12.28 (SD = 2.95), with a median score of 12. Using the categorization of the knowledge-practice scores explained in the median split method, we formed two groups of adequate and inadequate knowledge-practice of parents respectively. Out of the 528 parents who answered the questionnaire, 66.1% of the study population was found to have adequate knowledge-practice scores, whereas 33.9% were found to have inadequate knowledge-practice scores.

Table 1 shows the 20 statements of knowledge and practice. This scale consisted of two parts. The first part contained 10 statements of knowledge (1–10) and the second contained 10 statements of practice (11–20). The lowest correct answer (10.6%) was apparent in the question (8) related to the knowledge of vaccine storage, as in statements 2, 7, 18, and 20, for which the percentage of correct answers was 35.4%, 39%, 23.7%, and 40.2% respectively. The highest correct answer (96%) was apparent in the statement related to the practice of vaccine recommendation (12), as in statements 1, 6, 11, and 13, for which the percentage of correct answers was 84.7%, 81.6%, 93.9%, and 85.4%, respectively.

The study found a significant association of immunization completeness with total knowledge and practice groups (*p* < 0.05). A higher percentage of parents with adequate knowledge and practice were found for children with complete immunization (71.7%) and partial immunization (59.5) than other groups, as shown in Table 2.

**Table 2:** Association between immunization completeness and knowledge-practice groups

**Groups**	**Immunization completeness (%)**		**Total (%)**	**P**
	**Partial immunization**	**Complete immunization**		
**Knowledge groups**				0.001^a^
Inadequate	137 (56.6)	118 (41.3)	255 (48.3)	
Adequate	105 (43.4)	168 (58.7)	273 (51.7)	
**Total**	**242 (100%)**	**286 (100%)**	**528 (100%)**	

Chi-square test, ^a^*p* < 0.05.

The Mann–Whitney test was used to find differences in knowledge and practice scores between immunization completeness groups. Table 3 shows the differences in knowledge and practice scores among variable groups. Significant differences in the knowledge- practice scores were shown among immunization completeness groups.

**Table 3:** KP scores differences between immunization completeness groups

**Immunization completeness**	**N (%)**	**Mean**	**Median**	**P**
**Knowledge-practice scores**				0.001^a^
Complete immunization	286 (54.2)	12.69	13.0	
Partial immunization	242 (45.8)	11.8	12.0	

Mann–Whitney U test, ^a^*p* < 0.05.

## Discussion

According to the findings, the children were vaccinated as a result of various reasons: the parents had a good perception of vaccination benefits and risks; the parents thought that the immunization was mandatory; and/or the parents knew that immunization was required for school registration or day care attendance [29,30].

Many reasons were found for not vaccinating children or not completing the vaccination schedule; firstly, this may have been due to a lack of vaccination information among parents or health care providers. Inadequate information on vaccination status may lead to inappropriately timed or missed immunizations, resulting in decreased protection against diseases, increased side effects, and increased costs [31,32]. Secondly, this may have been due to the immunization card or clinical records not providing a clear and complete immunization record. The immunization card is very important for the immunization provider to be able to determine which vaccination is due on a child’s visit. In addition, the immunization card is important for parents to be able to determine or check their child’s immunization status [30,32].

Few studies have determined parental knowledge and practice related to childhood immunization and examined the association with children’s immunization status [33,34]. This study is the first to evaluate Arab and Iraqi parents' knowledge and practice and to determine the relationship of knowledge and practice of parents with immunization status of children younger than two.

The result of this study was similar to other findings in an Italian study [35] in which 57.8% of parents had adequate knowledge-attitude-practice (KAP), and is supported by a study in India [24] that found parental knowledge regarding vaccination adequate.

Although the situation in Iraq after 2003 was critical, causing some families to flee from one area to another area, and many changes in their immunization and health providers occurred, this study found that most Iraqi parents had adequate knowledge and good practice regarding child vaccination. This could be because of an increase in sources of vaccination and health information represented by television, the internet and other sources. Before 2003, many restrictions were imposed on the media, especially television and the internet, whereas an increase in the number of international medical and scientific TV channels, and an increase in internet users especially after 2003 are the important causes of the increase in parents' immunization practice and in immunization knowledge [36]. In addition, it should be highlighted that a difference in knowledge and practice scores does not imply a lack of intelligence in any of the parental groups. Historical insufficiency regarding vaccination knowledge practice and also variations in past education, health services and health education underlie the findings.

Most parents were in favour of immunization for children and thought that vaccination would prevent infectious diseases in the future, as shown in question 1 when more than 83% of parents gave the correct answer. The finding is similar to results in other studies [22,37] in which more than 90% of parents favoured child vaccination. Although most parents knew that vaccines prevent diseases, more than 60% did not know that vaccination was for all ages but only applied to children below school age (six years). This erroneous parental behaviour regarding vaccination age may have two causes; the general idea of Iraqi families that important and necessary vaccinations should be given before school age, and the low availability in health clinics and private pharmacies of vaccines appropriate for people older than six. This negative finding is inconsistent with the positive finding in another study [22] in which more than 70% of parents knew that vaccination was for all ages without exception.

Questions 3, 4, and 5 are related to vaccine types. Most parents (70.8%) knew that there were two different vaccine types. More than 35% of parents did not know what active vaccines were made of and 54% of parents did not know what the base of passive immunization is. Vaccines are mainly used for active immunization, especially those given to children under two, which is why the parents who knew about active immunization (64.6%) outnumbered the parents who knew about passive immunization (46%).

Severe allergic reaction, prolonged seizures, prolonged systemic steroid therapy or immunodeficiency disease are the most important contraindications of immunization [38]. The present study asked the parents about vaccine contraindications (question 6): although most parents (81.6%) thought that fever was the most important vaccination barrier but did not specify the degree (mild, moderate, high), fever ≥40.5°C is a factor to be taken into account but is not a contraindication according to immunization recommendations [38]. This finding was similar to other studies [39,40] in which the majority of parents had low knowledge regarding immunization contraindication and the parents stated that fever, allergy to egg protein, pregnant women and breastfeeding women are immunization contraindications.

The majority of parents gave incorrect answers (61% and 89.4%) to questions or statements related to vaccine storage and handling (Q.7 and Q.8), possibly because of the level of parents' education or type of employment. Most of the parents who gave correct answers might have medical or scientific bachelor's degrees or higher.

Approximately 65% of parents were able to answer correctly the question related to vaccination schedules (Question 9) and identify the timing of seven vaccine doses routinely given to children younger than two. This is consistent with results reported in a previous study [41] in which an immunization knowledge questionnaire administered at the Children's Hospital in Boston produced a mean score of 76% on questions relating to the schedule and administration of childhood vaccines.

In question 10, more than 55% of parents answered correctly that vaccination is potentially harmful. It shows that most of the parents acknowledged vaccine side effects. Parents in this study seem to be more aware of vaccine risk/benefit than parents in another study [22] in which 36.1% of parents believed that vaccines could be potentially harmful after vaccination.

Positively finding was found in the first and second questions in the part of practice questionnaire (Q11, Q12), approximately 94% of parents favored vaccination for their children and 96% of parents recommended immunization to other parents [42]. That means the parents have good immunization practice and adequate information about the benefits of vaccination in the future and they have great trust in the immunization programme. This finding is similar to other findings in a study in Pakistan [22] in which 96% of parents were in favour of vaccination for their children, but the percentage of parents (57.7%) that recommended the vaccination to others was lower than the percentage of Iraqi parents, and this difference could be related to the different environment and socioeconomic status prevailing in each country.

In addition, most parents correctly answered question 13, which related to the first immunization dose in the first week of life; the highest frequency of parents (451 parents) was referred to a good experience in immunization field and this was drawn from older children's experience or that of friends or relatives.

The majority received information about vaccines from physicians and other medical staff. Further work is needed to increase physicians' and immunization providers' knowledge in this area [43]. In this study, parents are noted as one of the important sources of immunization information but their immunization knowledge needs to be strengthened. According to question 14, 77.5% of parents were informed about vaccination and these parents are one of immunization’s information sources, a finding similar to another study [22] in which most parents were informed about immunization and noted as another information source.

According to the parents’ answers to questions 15, 16, 17, and 18, about 59.5% of parents collected the information from literature by reading, approximately 70% of parents preferred information from the television, less than 41% of parents heard about vaccination from radio, and about 25% of parents received vaccination information from the internet. The above results suggest television is the best source for immunization information because television is freely available at home and it is more convenient for parents to watch medical programmes than use the internet: not all parents know how to use the internet or obtain information by reading. However, this result was inconsistent with other studies [22,44] which consider internet as the main source of information on vaccination.

The last two questions (Questions 19 and 20) in the practice questionnaire assessed and evaluated the antenatal clinic and maternity hospital as immunization information providers. The result showed that parents received the information from clinics (65.9%) more than from hospitals (40.2%). Parents visit health clinics for routine child health care but visit the hospital for child delivery only and do not visit it again. Other studies in India and Bangladesh [45,46] showed that nurses and other medical workers were considered the main sources of immunization information for mothers, and other study in India found that mothers received weak information from medical staff owing to the poor communication between them [47].

The levels of KP among parents were positively associated with their children’s immunization rate was found in this study. The finding of this study is consistent with other studies’ findings [48,49] in which knowledge regarding vaccination is correlated with immunization rates. In addition, the results are supported by an Italian study of mothers [35] which showed that mothers’ lack of knowledge regarding vaccination is an important reason for failure to complete the immunization schedule. Another study [34] revealed that parents' good knowledge could not explain the low immunization rate of their children.

## Conclusions

There is a need to increase awareness and knowledge about the benefits and importance of vaccination, as well as the harmful consequences of non-complete immunization. A planned educational programme is needed; the educational level of the parents needs to be taken into consideration when the programme is planned, especially as regards those with a lower educational level.

## Competing interest

We would like to declare that there was no conflict of interest in conducting this research.

## Authors’ contributions

All authors have made substantial contributions to the conception of the study, drafting the article, and final approval of the version to be submitted. OQ, MB, and HQ conceived and designed the study. OQ and MR did the electronic search for the relevant articles and drafted the manuscript. MR and RM analyzed the data. OQ and SJ revised and edited the manuscript. RM and SJ prepared the manuscript for publication. All authors have read and approved the final submitted manuscript.

## Pre-publication history

The pre-publication history for this paper can be accessed here:

http://www.biomedcentral.com/1471-2431/14/20/prepub
